# Association between obesity and metabolic co-morbidities among children and adolescents in South Korea based on national data

**DOI:** 10.1186/1471-2458-14-279

**Published:** 2014-03-25

**Authors:** Hyunjung Lim, Hong Xue, Youfa Wang

**Affiliations:** 1 Department of Medical Nutrition, Graduate School of East-West Medical Science, Kyung Hee University, Yongin-si, Gyeonggi-do, Republic of Korea; 2 Research Institute of Medical Nutrition, Kyung Hee University, Seoul, Republic of Korea; 3 Johns Hopkins Global Center on Childhood Obesity, Department of International Health, Johns Hopkins Bloomberg School of Public Health, Baltimore, MD, USA; 4 Department of Epidemiology and Environmental Health, School of Public Health and Health Professions, University at Buffalo, State University of New York, Buffalo, NY, USA

**Keywords:** Child, Adolescents, Body mass index, Obesity, Metabolic co-morbidity, Metabolic syndrome, South Korean, Waist

## Abstract

**Background:**

Childhood obesity has become a serious public health threat worldwide due to its many short- and long-term adverse health effects. We assessed the association between weight status and metabolic co-morbidities among South Korean children using nationally representative data.

**Methods:**

Data from the 2007-2008 Korea National Health and Nutrition Examination Surveys for 1,526 children aged 10-19 years were used. Logistic regression models were fit to examine the association between weight status [overweight, 85^th^ percentile ≤ BMI <95^th^ percentile; obese, BMI ≥95^th^ percentile; and central obesity, waist circumference ≥90^th^ percentile, based on 2007 Korean National Growth Charts] and metabolic outcomes.

**Results:**

More obese children had metabolic co-morbidities than normal-weight children (P < 0.05). Boys had higher means BMI than girls, WC, and BP, while girls had higher means of total blood cholesterol and HDL-cholesterol (P < 0.05). Attributable risk of metabolic syndrome was high due to overweight and obesity: 91.1% for central obesity and 29.6% for high TG. Obese children had increased risk of having high BP (adjusted odds ratio (OR): 1.90; 95% CI: 1.05-3.45), dyslipidemia (OR: 6.21; 95% CI: 3.59-10.75), high TG (OR: 6.87; 95% CI: 4.05-11.64), low HDL (OR: 4.46; 95% CI: 2.23-8.89), and ≥2 co-morbidities (OR: 26.97; 95% CI: 14.95-48.65) compared to normal-weight subjects, while the associations between weight status and metabolic outcomes were stronger in boys.

**Conclusions:**

Obesity was strongly associated with metabolic co-morbidities in South Korean children.

## Background

Worldwide prevalence of childhood obesity has increased remarkably over the past three decades [1]. Overweight or obese children are at a high risk for developing long-term chronic conditions, including high blood pressure (BP), elevated blood glucose, dyslipidemia, and metabolic syndrome (MetS) [2,3], which are metabolic co-morbidities of obesity.

As South Korea became industrialized, energy intake and sedentary activities (e.g., screen time) have increased. Based on the 2011 Korea national health statistics, among Korean adults aged 30 and older, the prevalence of obesity (BMI ≥ 30 kg/m^2^), hypertension, diabetes, hypercholesterolemia, and hypertriglyceridemia is 34.2%, 30.8%, 10.5%, 14.5%, and 16.5%, respectively. The rates have been increasing [4].

The prevalence of childhood obesity doubled from 5.4% in 1998 to 10.8% in 2008 in South Korea, although it is still low compared to Western countries [5,6]. Moreover, among Korean adolescents body weight status misperception (51.6% of boys; 48.6% of girls) is common based on the 2009 nationally representative data. It is strongly associated with poor eating behaviors, and stress or depressed moods [7]. However, little is known about the association between weight status and metabolic co-morbidities among children in South Korea [6]; previous related studies were mainly based on data that is 10 years old, and the gender-age specific association between metabolic co-morbidities and weight status has not been sufficiently examined.

To fill this gap, this study assessed the association between weight status and metabolic co-morbidities by gender among children aged 10-19 years in South Korea using nationally representative data. We also investigated the aspect of metabolic co-morbidities in overweight/obese children. The findings may help guide future screening of high-risk groups and guide related interventions.

## Methods

### Study population and database

We used data from the fourth Korea National Health and Nutrition Examination Surveys (KNHANES IV, 2007-08). KNHANES is a series of population-based, cross-sectional surveys that selects a representative group by using a stratified, multistage sampling design according to geographic area, age, and gender. Since 2007, the KNHANES has become a year-round investigation with rolling survey sampling. Stratification was conducted based on the country's 29 areas, including 11 metropolitan cities and provinces, the administrative unit, and the dwelling type. The instruments included a health interview survey, a health examination survey, and a nutrition survey. The health interview survey collected information regarding socio-demographic characteristics. Anthropometry, BP measures and laboratory tests of the subjects were obtained by direct health examination in a mobile examination center. Detailed descriptions of the study design and data collection have been published [8]. The KNHANES IV was approved by the Korea Centers for Disease Control & Prevention Institutional Review Board.

Blood sample laboratory tests among children under 10 years of age were not available. We focused on a sample of 3,618 children (1,658 boys and 1,510 girls) aged 10-19 years with complete demographic data. After excluding 645 subjects without body mass index (BMI) measures and 1,447 subjects without metabolic co-morbidities parameters such as waist circumference (WC), BP, glucose, triglyceride (TG) and total cholesterol (TC), and high-density lipoprotein cholesterol (HDL-C), we had a final sample of 1,526 subjects (821 boys and 705 girls) for our analyses.

### Data collection and key measurements

#### Anthropometric measures

Standing height (seca 225, SECA, Germany) and weight (GL-6000-20, CASKOREA Co., Ltd., Korea) were obtained using standardized techniques and equipment. WC was measured at the midpoint between the subcostal bottom and the top of the iliac crest using a fiberglass tape (seca 200, SECA, Germany).

#### Blood pressure (BP)

Three BP determinations were obtained by standard methods with the subject in a sitting position using a mercury sphygmomanometer. After rest while seated, BP was measured 3 times at 30 second intervals. The average of the second and third measurements was used in our analysis.

#### Laboratory tests

All blood specimen collection and processing instructions are described in the KNHANES Laboratory/Medical Technologists Procedures Manual [9]. Blood samples were collected in the morning after fasting for at least 8 hours. Fasting blood glucose was analyzed using a reaction between glucose and ATP catalyzed by the enzyme hexokinase. The concentrations of TG, TC, and HDL-C were also measured using an enzymatic method. Fasting blood glucose, TG, TC, and HDL-C levels were measured using an automated hematology analyzer (ADVIA 1650, Siemens, USA) in a central, certified laboratory. The low-density lipoprotein cholesterol (LDL-C) concentration was estimated by the Friedewald formula (LDL cholesterol = total cholesterol-HDL cholesterol-triglycerides/5) [10].

#### Definition of weight status

To define weight status, BMI was calculated based on measured weight and height (BMI = weight (kg)/squared height (m^2^)). We used the 2007 Korean National Growth Charts [11]. Subjects were classified as a) normal weight, 5^th^ percentile ≤ BMI < 85^th^ percentile; b) overweight, 85^th^ percentile ≤ BMI < 95^th^ percentile; c) obese, BMI ≥ 95^th^ percentile [11].

Central obesity is defined as WC ≥ 90th percentile for age and gender based on 2007 Korea Growth Charts [11] in children aged 10 to < 16 yrs., and ≥ 90 cm for men and ≥ 85 cm for women as the Korean-specific criterion [12] in adolescents aged ≥ 16 yrs.

#### Definition of metabolic co-morbidities

The definition of metabolic co-morbidities for our study was as follows:

1) Prehypertension and hypertension [13]: systolic BP/diastolic BP ≥ 90th percentile for age, gender, and height based on 2007 Korea Growth Chart [11]

2) High glucose [14]: fasting blood glucose ≥ 100 mg/dL [14]

3) Dyslipidemia [15]: high TG, high TC, high LDL-C, or low HDL-C [15]

i) High TG: Triglyceride > 150 mg/dL

i) High TC: Total cholesterol > 200 mg/dL

i) LDL-cholesterol > 130 mg/dL

i) HDL-cholesterol < 35 mg/dL

4) Metabolic syndrome: Having central obesity plus ≥ 2 of the following criteria based on the 2007 pediatric International Diabetes Federation (IDF) definition [16]

i) elevated BP: systolic BP ≥ 130 or diastolic BP ≥ 85 mm Hg, or treatment of previously diagnosed hypertension

i) elevated fasting plasma glucose: ≥ 100 mg/dL (5.6 mmol/L), or previously diagnosed type 2 diabetes

i) elevated TG level: ≥ 150 mg/dL (1.7 mmol/L), or specific treatment for this lipid abnormality

i) reduced HDL cholesterol: < 40 mg/dL (1.03 mmol/L) in children aged 10-16 yrs and boys aged 16-19 yrs, and < 50 mg/dL (1.29 mmol/L) in girls aged 16-19 yrs, or specific treatment for this lipid abnormality

### Statistical analysis

Our key outcome variables were the presence of metabolic co-morbidities (e.g., central obesity, high BP, high glucose, dyslipidemia, metabolic syndrome). The key exposure categorical variable was weight status classified as normal weight, overweight, obesity, and central obesity. Analysis stratified by age and gender was conducted. The complex sample design was taken into account in the analysis using STATA release 11.0 survey-related commands, to give nationally representative estimates and correct estimates of the related variances.

First, we examined the gender and weight status differences in metabolic co-morbidities. Between-group differences in means were tested using *t* test and ANOVA, and the prevalence differences of metabolic co-morbidities were tested using *χ*^2^ tests. We also tested the trends across weight status (normal weight, overweight, obesity) and calculated population attributable risk (PAR, %) [17].

Then, to examine the association between weight status and metabolic co-morbidities (outcome variables), we fitted logistic regression models, controlling for socioeconomic and other environmental factors, including urban-rural residence region, household income as the quartiles of average household monthly income, and menarche status (for girls).

## Results

### Prevalence of metabolic co-morbidities by gender and weight status

Most participants (81.6%) lived in urban areas. Age distribution, urban-rural residence region, and household income did not differ significantly across the three categories of weight status. Table 1 shows the distribution of metabolic outcomes by gender and weight status. Boys suffered worse metabolic outcomes in terms of high WC level and elevated BP level compared to girls, whereas girls had higher mean TC and HDL-C levels (P < 0.05) than boys. Obese children had significantly higher means for most metabolic outcomes (e.g., BMI, WC, BP, TG, TC, LDL-C, atherogenic index = defined as log (TG/HDL-C), waist-to-height ratio); while, mean HDL-C level was the highest among normal-weight children (all P < 0.05).

**Table 1:** **Means and proportions of metabolic outcomes by gender and weight status among children aged 10-19-y-old in South Korea: KNHANES 2007-2008**^**1**^

	**By gender**						**By weight status**^**2,3,4**^					
	**Total**		**Boys**		**Girls**		**Normal weight**		**Overweight**		**Obese**	
	**(n = 1526)**		**(n = 821)**		**(n = 705)**		**(n = 1147)**		**(n = 194)**		**(n = 90)**	
	**Mean**	**SE**	**Mean**	**SE**	**Mean**	**SE**	**Mean**	**SE**	**Mean**	**SE**	**Mean**	**SE**
**Anthropometry**												
Body mass index (kg/m^2^)	20.5	0.1	21.0	0.2	20.0	0.1^5^	19.6	0.1^c^	24.7	0.1^b^	28.9	0.4^a^
Waist circumference (cm)	70.0	0.3	72.2	0.5	67.3	0.4^5^	67.4	0.3^c^	80.3	0.6^b^	90.3	1.5^a^
Systolic blood pressure (mmHg)	104.0	0.4	106.5	0.5	101.0	0.4^5^	102.9	0.4^c^	108.0	0.9^b^	112.9	1.5^a^
Diastolic blood pressure (mmHg)	65.4	0.4	66.7	0.5	64.0	0.5^5^	65.0	0.4^b^	67.6	0.9^a^	67.6	1.5^a^
**Laboratory tests**												
Triglycerides (mg/dL)	90.8	1.9	89.6	2.7	92.1	2.4	82.9	1.7^c^	119.3	8.4^b^	142.0	10.6^a^
Total cholesterol (mg/dL)	157.8	0.9	155.5	1.2	160.5	1.1^5^	155.6	1.0^c^	167.0	2.2^b^	175.9	3.0^a^
LDL-cholesterol (mg/dL)	89.9	0.8	88.8	1.1	91.2	1.1	88.5	0.9^c^	96.2	2.1^b^	105.2	3.2^a^
HDL-cholesterol (mg/dL)	49.7	0.5	48.8	0.6	50.8	0.6^5^	50.6	0.6^a^	47.0	0.9^b^	42.2	1.4^c^
Fasting blood glucose (mg/dL)	89.3	0.4	89.6	0.5	88.8	0.4	89.0	0.3^b^	91.6	1.3^a^	89.8	1.0^ab^
**Atherogenic index**^**7**^	-0.3	0.0	-0.3	0.0	-0.3	0.0	-0.4	0.0^c^	-0.1	0.1^b^	0.3	0.2^a^
**Waist-to-height ratio**	0.4	0.0	0.4	0.0	0.4	0.0^5^	0.4	0.0^c^	0.5	0.0^b^	0.6	0.0^a^
	%		%		%		%		%		%	
**Obesity status**												
Central obesity, %^8^	8.1		8.9		7.2		0.9		20.8		77.6^6^	
Overweight and obesity, %^2^	19.0		21.0		16.7							
**Cadiometabolic risk factors**												
Metabolic syndrome, %^6^	1.9		2.4		1.3		0.0		3.2		24.7^6^	
High blood pressure, %^7^	17.5		20.0		14.7^6^		15.9		22.6		28.4^6^	
High glucose, %^8^	6.7		7.1		6.3		6.5		8.0		9.2	
Dyslipidemia, %^9^	21.2		21.8		20.4		16.2		38.2		55.4^6^	
High triglycerides, %^9^	10.2		10.0		10.4		6.9		21.5		33.1^6^	
High total cholesterol, %^9^	6.2		5.6		6.8		5.0		10.8		14.4^6^	
High LDL-cholesterol, %^9^	5.6		5.2		6.0		4.4		7.8		16.3^6^	
Low HDL-cholesterol, %^9^	7.9		9.2		6.3		6.1		12.0		23.2^6^	
**No. of metabolic comorbidities, %**^**10**^												
0	61.4		60.8		62.2		69.2		36.1		8.9^6^	
1	28.4		28.8		27.9		25.2		46.4		30.8	
≥ 2	10.2		10.4		10.0		5.7		17.5		60.3	

*Abbreviations*: *KNHANES* Korea National Health and Nutrition Examination Survey, *LDL-cholesterol* Low density lipoprotein cholesterol, *HDL-cholesterol* high density lipoprotein cholesterol.

^1^Estimates shown as mean and standard error or %.

^2^Defined using 2007 Korea growth chart, Normal weight (5th percentile ≤ BMI < 85th percentile); overweight (85th percentile ≤ BMI < 95th percentile); obesity (BMI ≥ 95th percentile).

^3^P-trend <0.05 across weight status.

^4^Letters with different superscripts by weight status are significantly different at P<0.05 by Tukey’s multiple range test.

^5^P < 0.05 for between group comparisons.

^6^P < 0.05 for between group comparisons using χ^2^ tests.

^7^Atherogenic index = log (triglyceride/HDL-cholesterol).

^8^Definitions of metabolic co-morbidities are provided in Methods.

^9^Among elevated waist circumference, elevated triglycerides, reduced HDL cholesterol, elevated blood pressure, elevated fasting plasma glucose based on the 2007 pediatric IDF definition.

Regarding prevalence of metabolic abnormalities, obese children had a high prevalence of MetS, central obesity, high BP, dyslipidemia and all individual blood lipid profiles (P < 0.05), except for the prevalence of high glucose (P > 0.05). In addition, the proportion of children with 2 or more metabolic co-morbidities is higher among overweight and obese children than their normal weight counterparts (5.7% in normal weight vs. 17.5% in overweight vs. 60.3% in obese children, P < 0.05).

### Attributable risk of metabolic comorbidities to overweight or obesity by gender

**Figure 1:**
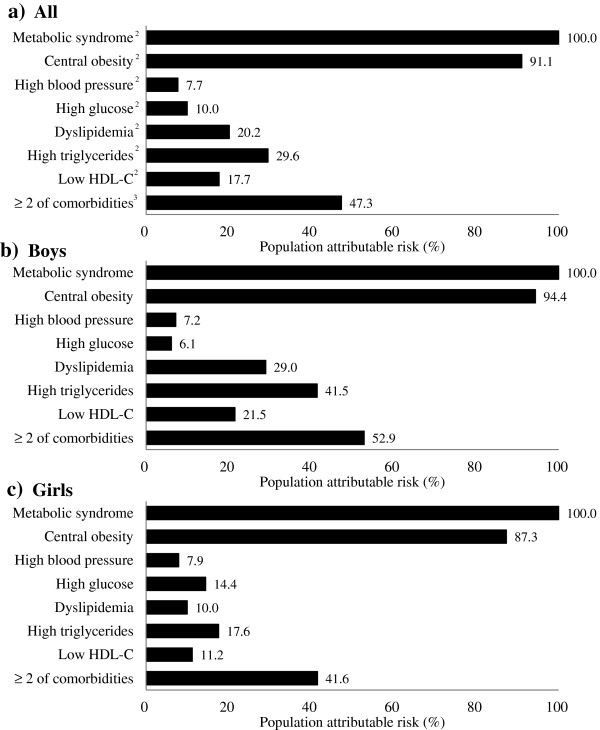
**Risk of metabolic comorbidities attributable to overweight and obesity among children aged 10-19-y-old in South Korea: KNHANES 2007-2008.** KNHANES, Korea National Health and Nutrition Examination Survey; HDL-C, high density lipoprotein cholesterol. ^1^Unadjusted population attributable risks estimated based on the method developed by Fleiss [15]. ^2^Definitions of metabolic co-morbidities are provided in Methods. ^3^Among elevated waist circumference, elevated triglycerides, reduced HDL cholesterol, elevated blood pressure, elevated fasting plasma glucose based on the 2007 pediatric IDF definition.

Overall, the risk of metabolic co-morbidities attributable to overweight or obesity was high for MetS (100.0%), central obesity (91.1%), and ≥ 2 metabolic co-morbidities (47.3%); modest for high TG (29.6%), dyslipidemia (20.2%), and low HDL (17.7%); and relatively low for high BP (7.7%) and high glucose (10.0%) (Figure 1). Boys had high attributable risk rates of metabolic co-morbidities compared to girls, except for the attributable risk due to high glucose (6.1% in boys vs. 14.4% in girls).

### Association between weight status and metabolic co-morbidities by gender

Table 2 shows the associations between weight status and metabolic co-morbidities. After adjusting for age, gender, income, urban-rural residence, and menarche status (only for girls), the estimates showed that: 1) obese children were more likely to have high BP (OR, 1.90; 95% CI, 1.05-3.45), dyslipidemia (OR, 6.21; 95% CI, 3.59-10.75), high TG (OR, 6.87; 95% CI, 4.05-11.64), high TC (OR, 2.80; 95% CI, 1.10-5.62), high LDL (OR, 3.81; 95% CI, 1.87-7.80), low HDL (OR, 4.46; 95% CI, 2.23-8.89), and ≥ 2 co-morbidities (OR: 26.97; 95% CI: 14.95-48.65) than normal-weight subjects (P < 0.05); and 2) centrally obese children had higher risks of having dyslipidemia (OR, 3.87; 95% CI, 2.45-6.13), other blood lipid profiles (OR, 2.04-4.12, P < 0.05), and ≥ 2 co-morbidities (OR: 19.90; 95% CI: 12.09-32.76) than children who were not centrally obese.

**Table 2:** **Associations (OR and 95% CI) between overweight and obesity and individual metabolic comorbidities among children aged 10-19-y-old in South Korea: KNHANES 2007-2008**^**1,2**^

	**Model 1**	**Model 2**	**Model 3**	**Model 4**	**Model 5**	**Model 6**	**Model 7**	**Model 8**
	**High blood pressure**^**3**^	**High glucose**^**4**^	**Dyslipidemia**^**5**^	**High triglyceride**^**4**^	**High cholesterol**^**5**^	**High LDL-C**^**5**^	**Low HDL-C**^**5**^	**≥2 of MetS components**^**6**^
**In boys and girls**								
Overweight^7^	1.46 (0.94-2.28)	1.27 (0.64-2.51)	3.36 (2.35-4.81)	3.80 (2.29-6.30)	2.33 (1.27-4.30)	1.91 (0.98-3.74)	2.17 (1.14-4.11)	3.83 (2.25-6.52)
Obese^7^	1.90 (1.05-3.45)	1.54 (0.68-3.47)	6.21 (3.59-10.75)	6.87 (4.05-11.64)	2.80 (1.10-5.62)	3.81 (1.87-7.80)	4.46 (2.23-8.89)	26.97 (14.95-48.65)
Central obesity^8^	1.15 (0.69-1.90)	1.65 (0.82-3.35)	3.87 (2.45-6.13)	4.12 (2.67-6.37)	2.04 (1.07-3.90)	3.01 (1.58-5.74)	3.78 (1.81-7.90)	19.90 (12.09-32.76)
**In boys**								
Overweight^7^	1.31 (0.78-2.21)	1.21 (0.49-2.99)	5.00 (3.07-8.13)	6.28 (3.11-12.7)	2.91 (1.30-6.49)	2.65 (1.16-6.03)	2.64 (1.25-5.57)	5.31 (2.52-11.18)
Obese^7^	2.32 (1.08-4.98)	1.18 (0.31-4.45)	10.10 (4.84-21.07)	11.50 (5.24-25.02)	5.17 (1.78-14.95)	6.50 (2.66-15.91)	5.49 (2.42-12.42)	36.30 (16.93-77.84)
Central obesity^8^	1.42 (0.75-2.68)	1.04 (0.32-3.34)	5.32 (3.02-9.37)	5.35 (2.83-10.10)	3.65 (1.59-8.35)	5.80 (2.66-12.67)	3.65 (1.65-8.07)	23.59 (12.16-45.76)
**In girls**								
Overweight^7^	1.55 (0.70-3.43)	0.83 (0.28-2.51)	1.93 (1.05-3.53)	1.95 (0.86-4.41)	2.05 (0.72-5.90)	1.68 (0.48-5.92)	1.66 (0.55-5.05)	2.69 (1.18-6.14)
Obese^7^	0.99 (0.31-3.17)	1.71 (0.57-5.09)	2.37 (1.20-4.71)	3.04 (1.38-6.71)	0.75 (0.15-3.85)	1.30 (0.34-4.97)	1.64 (0.50-5.36)	13.84 (6.45-29.70)
Central obesity^8^	0.64 (0.24-1.69)	3.24 (1.37-7.64)	2.13 (1.14-3.95)	2.87 (1.39-5.91)	0.77 (0.21-2.88)	1.10 (0.36-3.38)	2.66 (0.94-7.55)	16.79 (8.51-33.11)

*Abbreviations*: *KNHANES* Korean National Health and Nutrition Examination Survey, *OR* odds ratio, *CI* confidence interval.

^1^Each logistic regression model adjusted for age, gender, income, and urban-rural residence; among girls, further controlled for menarche status.

^2^Normal weight (5th percentile ≤ BMI < 85th percentile) group was the reference group in each model.

^3^Systolic blood pressure/diastolic blood pressure ≥ 90th percentiles of 2007 Korean CDC growth chart.

^4^Fasting blood glucose ≥ 100 mg/dL.

^5^Triglyceride > 150 mg/dL or Total cholesterol > 200 mg/dL or LDL-cholesterol: > 130 mg/dL or HDL-cholesterol: < 35 mg/dL.

^6^Among elevated waist circumference, elevated triglycerides, reduced HDL cholesterol, elevated blood pressure, elevated fasting plasma glucose based on the 2007 pediatric IDF definition.

^7^By BMI percentile of 2007 Korea growth chart, overweight (85th percentile ≤ BMI < 95th percentile); obesity (BMI ≥ 95th percentile).

^8^Waist circumference ≥90th percentile for age and gender based on 2007 Korean CDC growth chart.

Our results suggested strong associations between weight status and metabolic outcomes in boys, in particular. In contrast, co-morbidities such as high BP, high TC, high LDL, and low HDL were not significantly associated with weight status in girls. However, centrally obese girls seemed to have a higher risk of high glucose (OR: 3.24; 95% CI: 1.37-7.64) than girls with normal WC.

## Discussion

We examined the association between weight status and metabolic co-morbidity among children by gender using the 2007-08 nationally representative data in South Korea. Obese, including centrally obese, children had a higher prevalence of metabolic co-morbidities than normal-weight children. Overweight or obese children had also higher attributable risks of MetS, central obesity, and higher TG. Our results showed strong associations between weight status and metabolic outcomes for boys, in particular. Hyperglycemia was a significantly higher risk outcome among centrally obese girls only. Our findings suggest that dyslipidemia, in particular high TG, was a key co-morbidity, which is affected by increased body weight.

Overall prevalence of MetS in our participants (1.9%) was similar to a previous study based on the 2005 KNHANES (1.8%) [18]. Korean adolescents had a slightly higher MetS prevalence based on the IDF criteria than adolescents from some other Asian countries (e.g., 1.2% in Hong Kong Chinese [19], 1.5% in Asian Indian adolescents [20]), but a lower rate than US adolescents (5.5%) [21] and Finland adolescents (2.4%) [22].

Among overweight and obese children, the MetS prevalence was higher than the previous 2005 KNHANES results (among overweight: 3.2% in 2007-08 vs. 1.5%, among obese: 24.7% vs. 14.7%) [18]. This is likely due to their higher adiposity among the overweight and obese children over time. The MetS rate in obese South Korean children was higher than in those from other populations of children and adolescents, e.g., 19.4% among Norwegians [23] and 10.3% among obese Chinese [24]. However, it should be noted that these studies focused on slightly different age ranges and used different MetS diagnosis criteria.

Obese children had a higher prevalence of most metabolic co-morbidities than non-obese children. These findings were consistent with the data reported for several other populations such as Mexican [25], Iranian [26], US [27], German [28], and Chinese [29] children. For example, risk of MetS was 16 times higher in US overweight adolescents compared to those with normal weight (BMI < 85^th^ percentile) (14.5% vs. 0.9%) [27]. In a study from China, a slightly increasing trend of metabolic parameter levels was found at the 75^th^ percentile of BMI and a significant increase was found when BMI ≥ 85^th^ percentile [29].

Interestingly, in our subjects, the overall prevalence of elevated BP (prehypertension and hypertension) was 17.5% and 21.2% of dyslipidemia, higher than other Asian populations [30-33]. According to a school-based population study of 88,974 Chinese adolescents, the prevalence was 10.3% [30]. Prehypertension can be predictive of future hypertension [34]. Our findings are consistent with the observation that pediatric hypertension is increasing with the rising pediatric obesity epidemic [35]. In South Korea, unlike for adults, blood pressure measurement is not a component of regular health examinations for children, thus, future monitoring and prevention efforts are needed to address this high prevalence. In terms of dyslipidemia, the prevalences were 11.1% in Chinese [32] and about 11.9% in Thai children [33], although differences exist on the definition of dyslipidemia. Most studies regarding dyslipidemia indicated that overweight and obese children were more likely to have dyslipidemia [36,37]. However, there are no local, official criteria of dyslipidemia for Korean children.

Boys had higher metabolic outcomes such as BMI, WC, BP levels and waist-to-height ratios; whereas, girls had higher TC and HDL levels. These gender differences were consistent with previous findings in other studies among adolescents in China [32] and even young adults aged 15-39 years among Asian Indians [38]. Likewise, boys may be the more vulnerable than girls to the risks of childhood metabolic disorders. Studying the reasons for boys' higher risks could shed some light on why the metabolic disorders are generally on the rise. Based on the same KNHANES 2007-08 data, South Korean men aged 20-65 years suffered worse metabolic outcomes than women (all P < 0.05), e.g., MetS: 15.8% in men vs. 11.6% in women, and had a higher average number of metabolic disturbances [39].

In addition, our results suggest a stronger association between childhood obesity and high TG (OR = 5.3 in 1998 KNHANES [6] in vs. 6.9 in 2007-08 KNHANES, compared to children of normal weight). With few exceptions, the associations between overweight (including obese and centrally obese) and individual metabolic co-morbidities were stronger in boys than in girls. Boys are therefore a more vulnerable group regarding metabolic outcomes, which may then lead to high risk of heart disease and other chronic diseases in adulthood. Additionally, Korean boys underestimated their weight [40]; therefore, we should help them to achieve a healthy body weight.

Previous research has suggested that obese children tend to have a higher risk of hyperglycemia and impaired fasting glucose [41]. However, our estimates do not show a difference in fasting blood glucose level and proportion of high glucose by weight status. Although a previous Korean study suggested that hyperglycemia is the most common individual component of the MetS in Korean adolescents (approximately 30% in both 1998 and 2001) [18], we did not find the same trend. Further studies need to verify this discrepancy. Nevertheless, we found that centrally obese girls tend to have 3 times higher risk of having elevated blood glucose compared to non-centrally obese girls. Similar findings were reported in studies about adult women in Sweden [42] and China [43].

In the present study, overweight or obese children had high attributable risks of MetS and central obesity. High TG (around 30% attributable risk) follows, which confers the highest risk of individual co-morbidities. While there is no or a very low prevalence of metabolic co-morbidities among normal-weight children, obese children had a high prevalence of MetS and central obesity. At present, overall, Korean children have little understanding of MetS, central obesity, or dyslipidemia. The importance of related health education was undervalued. Therefore, education programs to improve children’s recognition of these health dangers and their possible outcomes are needed.

This study has several key strengths. First, it was based on a recent, nationally representative sample in South Korea, and our analysis took into account the complex sampling design effect to provide representative estimates. Moreover, we investigated several metabolic co-morbidities of overweight or obesity among children.

On the other hand, our study could not employ longitudinal models due to data limitations which preclude causal inference and the estimates may suffer from problems such as reverse causation. Then, we categorized several variables of anthropometric and biochemical measurements. Categorization is a common practice in biomedical research, which facilitates the interpretation of model parameters and provides meaningful estimates with important clinic and public health implications. This is in line with the aims and scope of this study. However, the limitations of categorization should also be noted because the information in the data is not fully utilized. Future research that examines relevant variables on a continuous scale is warranted and could potentially help provide more insight. In addition, subjects were excluded in the analysis if weight or metabolic comorbidities were not measured, which limited our sample size and could potentially introduce selection bias. However, we explored the possibility of potential selection bias and our analysis did not suggest significant difference between the study sample and the whole sample. Moreover, the KHANES is the only available data set that can be used to examine the levels of biochemical indicators and estimate the prevalence patterns of chronic conditions at a national level. However, despite of the limitations, such as missing values, existing studies indicate that KNHANES can still provide valid and reasonably representative estimates, e.g. the prevalence of metabolic disorders among children [6,18,21].

## Conclusions

Childhood obesity is becoming a serious public health problem in South Korea, as 19.0% of children were overweight or obese, and 8.1% had central obesity. The prevalence of elevated BP (17.5%) and dyslipidemia (21.2%) was high among children in South Korea. In addition, most of the obese children (91.1%) had at least one metabolic co-morbidity. MetS and dyslipidemia, especially high TG, were the key co-morbidities among obese children. Lipid profiles of South Korean children are affected by their weight status. Intervention programs are needed to prevent and control obesity and its co-morbidities, in particular targeting at obesity in children.

## Abbreviations

BMI: Body mass index; BP: Blood pressure; HDL-C: High-density lipoprotein cholesterol; IDF: International Diabetes Federation; KNHANES: The Korea National Health and Nutrition Examination Surveys; LDL-C: Low-density lipoprotein cholesterol; MetS: Metabolic syndrome; PAR: Population attributable risk; TC: Total cholesterol; TG: Triglyceride.

## Competing interests

The authors declare that they have no competing interests.

## Authors’ contributions

HL and YW had full access to the data in the study and take full responsibility for the integrity of the study. HL analyzed the data. HL and YW drafted the paper. HX revised the manuscript. Findings were jointly interpreted by all authors. All authors contributed to successive drafts. The authors read and approved the final manuscript.

## Pre-publication history

The pre-publication history for this paper can be accessed here:

http://www.biomedcentral.com/1471-2458/14/279/prepub
